# Afterload-related reference values for myocardial work indices

**DOI:** 10.1186/s12947-021-00253-2

**Published:** 2021-06-24

**Authors:** Qiancheng Li, Hui Wang, Haiyan Feng, Tingfan Wu, Ying Yang, Dongmei Gao, Lina Sun

**Affiliations:** 1 Department of CT, Jilin Province FAW General Hospital, Changchun, China; 2 Department of Ultrasound, China-Japan Union Hospital of Jilin University, Changchun, China; 3 GE Healthcare, Clinical Education Team(CET), Pudong New Town, Shanghai, China

**Keywords:** Myocardial work, Afterload, Reference values

## Abstract

**Background:**

The novel noninvasive pressure-strain loop (PSL) is a reliable tool that reflects myocardial work (MW). Systolic blood pressure (SBP) is the only independent factor for MW indices. However, afterload-related reference values have not been previously reported. The aim of the present study was to establish reference values for MW parameters by wide range SBP grading.

**Methods:**

We prospectively selected healthy individuals and subjects with SBP ≥ 140 mmHg at the time of study without myocardial remodeling. MW parameters were collected and the reference values achieved were grouped by SBP in 10-mmHg.

**Results:**

Significant differences were noted among the SBP-groups for global work index (GWI) and global constructive work (GCW). The majority of statistical comparisons of the differences in GWI and GCW were significant at each SBP-group. With SBP ranging from 90 to 189 mmHg, the parameters GWI and GCW tended to increase linearly with afterload. Overall, the global wasted work (GWW) tended to rise as SBP was increased, but not all of the differences noted in GWW were significant for each SBP-group. Global work efficiency (GWE) remained stable across all SBP-groups, with the exception of a slight drop noted when it exceeded 160 mmHg.

**Conclusions:**

The amount of MW but not the work efficiency varied greatly according to the different afterload. This finding cannot be ignored during clinical research or diagnosis and afterload-related reference values are required to make a reasonable judgment on the myocardial function.

**Supplementary Information:**

The online version contains supplementary material available at 10.1186/s12947-021-00253-2.

## Background

The novel noninvasive pressure-strain loop (PSL) is a reliable tool to reflect myocardial work (MW) in a variety of hemodynamic states by comparison with invasive experimental and clinical studies. It has shown a strong correlation with regional myocardial glucose metabolism by positron emission tomography (PET) [1–4].

**Fig. 1:**
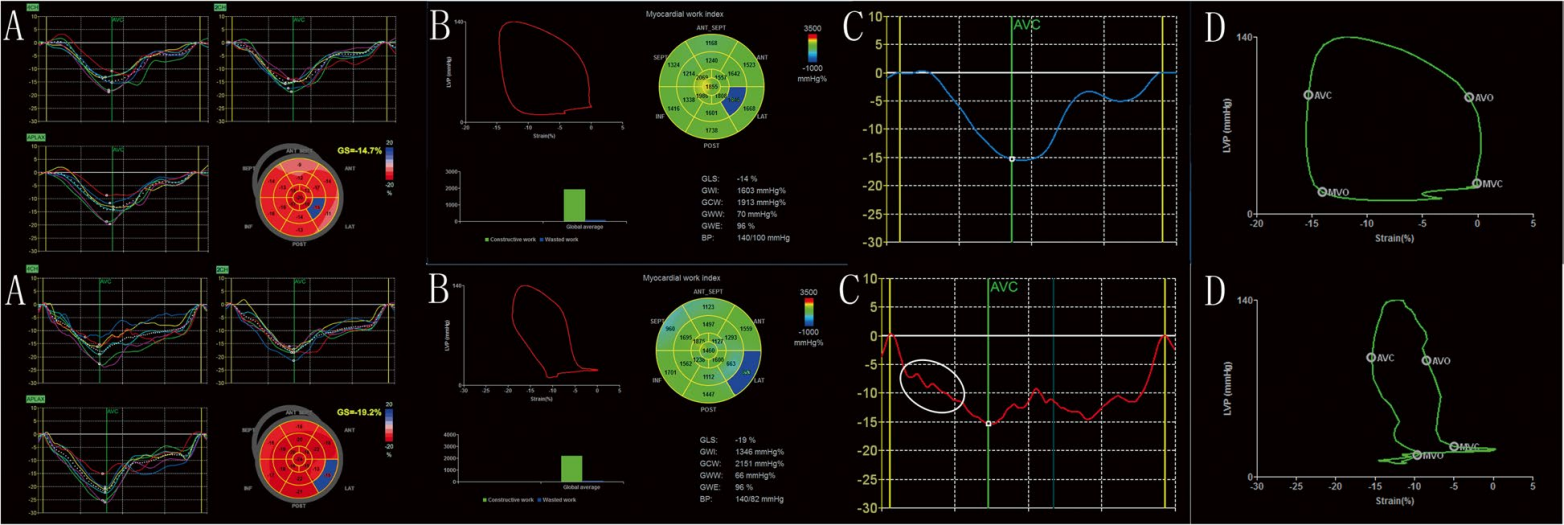
Top line: data from a patient after adrenal tumor surgery with a history of hypertension (SBP > 180 mmHg) for more than 2 years. Bottom line: data from a patient with coronary heart disease. Coronary angiography demonstrated stenosis in the middle left circumflex branch (80%) and in the proximal right coronary artery (70%). (**A**, **B** in the top line) The patient's GLS value was -14% with SBP of 140 mmHg at the time of the study. The GWI was 1,603 mmHg%. (**A**, **B** in the bottom line) This patient exhibited a higher GLS value of -19% and a lower GWI (1,346 mmHg%) at the same SBP level than the previously mentioned subject. Figure Cs and Ds explained these results. (**C**, **D** in the top line) The peak LS value of the middle segment of the lateral wall was -15% and the MWI of the segment was 1,846 mmHg%. (**C**, **D** in the bottom line) Considerably lower MWI was noted in the basal segment of the lateral wall (853 mmHg%) at the same peak LS and at the same SBP level. (**C** in the bottom line) It demonstrated that the strain curve with apparent transient hypokinisis, akinesis and even paradoxical movement in mid-systole (white oval) led to much lower MWI. However, this information could not be reflected by the LS value. SBP, systolic blood pressure; GLS, global longitudinal strain; GWI, global work index; LS, longitudinal strain; MWI, myocardial work index

The PSL algorithm has two advantages over strain measurements in evaluating left ventricular function. Firstly, the main limitation of strain imaging is load dependency [5]. An increase in afterload can lead to decreased strain giving rise to misinterpretation of the true contractile function, which in turn leads to false conclusions with regard to reduced myocardial function. However, MW takes into account deformation as well as afterload, potentially offering incremental value to myocardial function assessment. This advantage was proved by a canine experiment where a substantial decrease in longitudinal strain (LS) occurred, whereas myocardial work index (MWI) was unaltered during aortic constriction [6]. Secondly, MW parameters are calculated as integration over time of the strain rate obtained by differentiating the strain curve multiplied by the instantaneous left ventricular pressure (LVP). LVP was estimated by a surrogate of the LVP curve. Therefore, MW indices were used to address all the changes noted in the myocardial strain along with LVP changing during the cardiac cycle and provide more comprehensive information for evaluating left ventricular (LV) function. By contrast, LS, the most commonly used strain index, is used to assess the peak systolic strain value, but not the process of obtaining the peak strain. It is well known that LVP changes over time in the cardiac cycle. Therefore, the same strain occurring at different time periods during systole may achieve different MW measurements. The identical peak LS value, which occurs in early or mid-or end-systole may not correspond to identical myocardial work. However, this information cannot be represented by LS, nor can it be reflected in case of transient hypokinisis, akinesis or even paradoxical movement, which can affect the MW. In other words, different patients may present significantly different MW values even if they have the same LS and the same systolic blood pressure (SBP) (Fig. 1).

Therefore, theoretically, MW is the most comprehensive indicator of left ventricular myocardial function. Previous studies have reported reference values for MW indices grouped by gender or age [7–9]. Despite these reference values, the wide variability of MW indices prevents the application of this technique in the routine clinical setting. Multivariable linear regression analysis for MW indices has shown that SBP rather than gender or age was the only independent factor following adjustment for confounders [7]. Therefore, it is not advisable to assess MW in the absence of SBP. In other words, a given MW level for a patient with SBP of 90 mmHg has a significantly different meaning than the same MW for a patient with SBP of 180 mmHg. Given the superiority of MW, an urgent need is required to establish reference values for MW parameters grouped by SBP. The present study recruited healthy subjects without hypertension and subjects in the early stage of hypertension without myocardial remodeling. To the best of our knowledge, the present study provided for the first time the reference values for MW indices based on SBP grading over a wide range to benefit the application of this new indicator in the routine clinical diagnosis.

## Methods

### Subjects

We prospectively selected subjects from healthy volunteers and participants who had received coronary tomography angiography (CTA) for health examination demonstrating no more than 50% coronary artery stenosis. The eligibility criteria for healthy subjects included the following: 1. normal 2-dimensional (2D) echocardiographic results without grade > 1 regurgitation [10] 2. no history of diabetes mellitus, hyperlipidemia or cardiovascular disease 3. (1) SBP < 140 mmHg at the time of study and no history of hypertension or (2) SBP ≥ 140 mmHg at the time of study with history of hypertension < 1 year and without any myocardial remodeling based on normal 2D echocardiographic appearance and no history of antihypertensive medication administration. The present study was approved by the local ethics committees. All participants provided informed consent prior to the examination.

### Echocardiography

Comprehensive transthoracic echocardiography (TTE) was performed according to the American Society of Echocardiography guidelines [11, 12] by an experienced sonographer using a Vivid E95 ultrasound system equipped with an M5S 3.5 MHz and a 4VC 3.3 MHz transducer (GE Vingmed Ultrasound, Horten, Norway). The patients were scanned in the left lateral decubitus position for optimal image quality.

### MW

MW was measured from the PSL area using commercially available software Package (EchopacVersion 203, GE), which was constructed from a surrogate of the LVP curve combined with LS acquired with speckle tracking echocardiography (STE), as proposed by Russell et al. [1]. 2D grayscale images from the apical four-chamber, two-chamber and long-axis views were acquired at 62–86 frames/sec to enable LS analysis. In particular, the mitral and aortic valves were clearly displayed on the long-axis view. Three consecutive cardiac cycles for each view were acquired. For strain analysis, endocardial borders of all LV segments were clearly visualized throughout the whole cardiac cycle to assure optimal wall detection and tracking control. In addition, LV ejection fraction (EF) was achieved from apical four-chamber and two-chamber. The LVP was estimated in a noninvasive manner using peripheral brachial SBP, which was measured immediately following TTE with the patient being still in the left lateral decubitus position. When synchronized and normalized with valvular timing events, a surrogate of LVP versus time was generated. The LV strain and pressure data were subsequently synchronized by alignment of valvular timing events, which were all set manually on the long-axis view [13]. Instantaneous MW was quantified as the strain rate obtained by differentiating the strain curve multiplied by the instantaneous LVP. This instantaneous work was then integrated over time during systole (time interval from mitral valve closure through to mitral valve opening). The work performed during shortening in systole adding negative work during lengthening in isovolumetric relaxation represented constructive work (CW) and work performed during lengthening in systole adding work performed during shortening in isovolumetric relaxation represented wasted work (WW). CW and WW were calculated for each LV segment, according to the 17-segment model. Global CW (GCW) and global WW (GWW) were calculated as the averages of the segmental values. Work efficiency (WE) was then expressed as CW/ (CW + WW) × 100% per segment and the global WE(GWE) as an average of all segmental values. The Global work index (GWI) was obtained as total work calculated from mitral valve closure to mitral valve opening, equal to the area of the PSL.

To assess the intra-observer and inter-observer reproducibility, 40 subjects were selected (5 random subjects from each SBP-group). For intra-observer variability, one set of images were collected in these individuals by an experienced physician (LN.S.). The time interval for the analysis of the same set of images in each subject should be at least one week and it was applied in a random order. For inter-observer variability, the other dataset was retained by another physician (HY.F.) using the same device in the same place. The images were analyzed by the same physician (HY.F.).

### Statistical analysis

The normality of the distribution of continuous variables was assessed by the Kolmogorov–Smirnov test. Continuous data are reported as mean ± standard deviation (SD) or median (interquartile range) as appropriate. The 95% confidence interval (95% CI) was calculated as mean ± 1.96 SDs for normally distributed continuous variables. The lowest (2.5th percentile) and highest (97.5th percentile) expected values for non-normally distributed continuous variables were estimated in 1,000 bootstrap samples to generate sampling distribution. The comparison across SBP groups was performed by the analysis of variance (ANOVA) for normally distributed variables with homogeneity of variance or by the Kruskal–Wallis test for variables without normal distribution or homogeneity of variance and specific group differences were tested by using a corrected alpha value < 0.002. Scatter diagrams of GWI and GCW with SBP were plotted with trend curves obtained from locally weighted regression. Intra-observer and inter-observer variability was assessed using the Bland–Altman analyses and paired-samples t test or Wilcoxon matched paired test were used to verify the significance of the bias. The SPSS statistical software (version 21.0; SPSS Inc, Chicago, IL, USA) and R studio (version 3.6.1; R studio, Boston, Massachusetts) were used for all analyses. *P* < 0.05 was considered to indicate significant differences.

## Results

Of the 248 subjects included in the present study, 18 were excluded from further analysis due to poor image quality. Finally, a total of 80 men and 150 women were included. The demographic data of the population listed in Table 1 are grouped by SBP in 10-mmHg subgroups.

**Table 1:** characteristics of the population

characteristic	SBP-groups(mmHg)							
	90–99 *n* = 16	100–109 *n* = 29	110–119 *n* = 34	120–129 *n* = 45	130–139 *n* = 35	140–149 *n* = 29	150–159 *n* = 20	≥ 160 *n* = 22
Men, n(%)	2(13%)	5(17%)	10(29%)	14(31%)	17(49%)	16(55%)	7(35%)	9(41%)
Age(years)	32 ± 9	39 ± 14	40 ± 12	39 ± 12	43 ± 14	49 ± 13	47 ± 11	44 ± 13
Height(cm)	162 ± 5	162 ± 5	164 ± 8	165 ± 8	167 ± 7	168 ± 9	167 ± 10	168 ± 8
Weight(kg)	57 ± 6	57 ± 8	63 ± 11	63 ± 10	70 ± 12	74 ± 13	67 ± 12	73 ± 12
Body surface area (m^2^)	1.60 ± 0.10	1.61 ± 0.11	1.68 ± 0.18	1.69 ± 0.16	1.79 ± 0.17	1.83 ± 0.20	1.75 ± 0.20	1.81 ± 0.18
Heart rate(min^−1^)	76 ± 7	77 ± 9	79 ± 8	74 ± 10	76 ± 9	77 ± 7	78 ± 8	80 ± 7
LVMi(g/m^2^)	72 ± 10	73 ± 14	74 ± 10	74 ± 11	76 ± 11	79 ± 10	83 ± 12	86 ± 11
Fasting glucose (mmol/L)	4.74 ± 0.59	5.02 ± 0.56	5.00 ± 0.55	4.80 ± 0.53	4.85 ± 0.56	4.81 ± 0.43	5.07 ± 0.45	5.04 ± 0.49
Total cholesterol (mmol/L)	3.73 ± 0.60	3.89 ± 0.56	3.98 ± 0.66	4.09 ± 0.61	4.06 ± 0.59	4.27 ± 0.60	4.07 ± 0.64	4.22 ± 0.51

Data are expressed as mean ± SD. *SBP* systolic blood pressure; *LVMi* left ventricular mass index

**Fig. 2:**
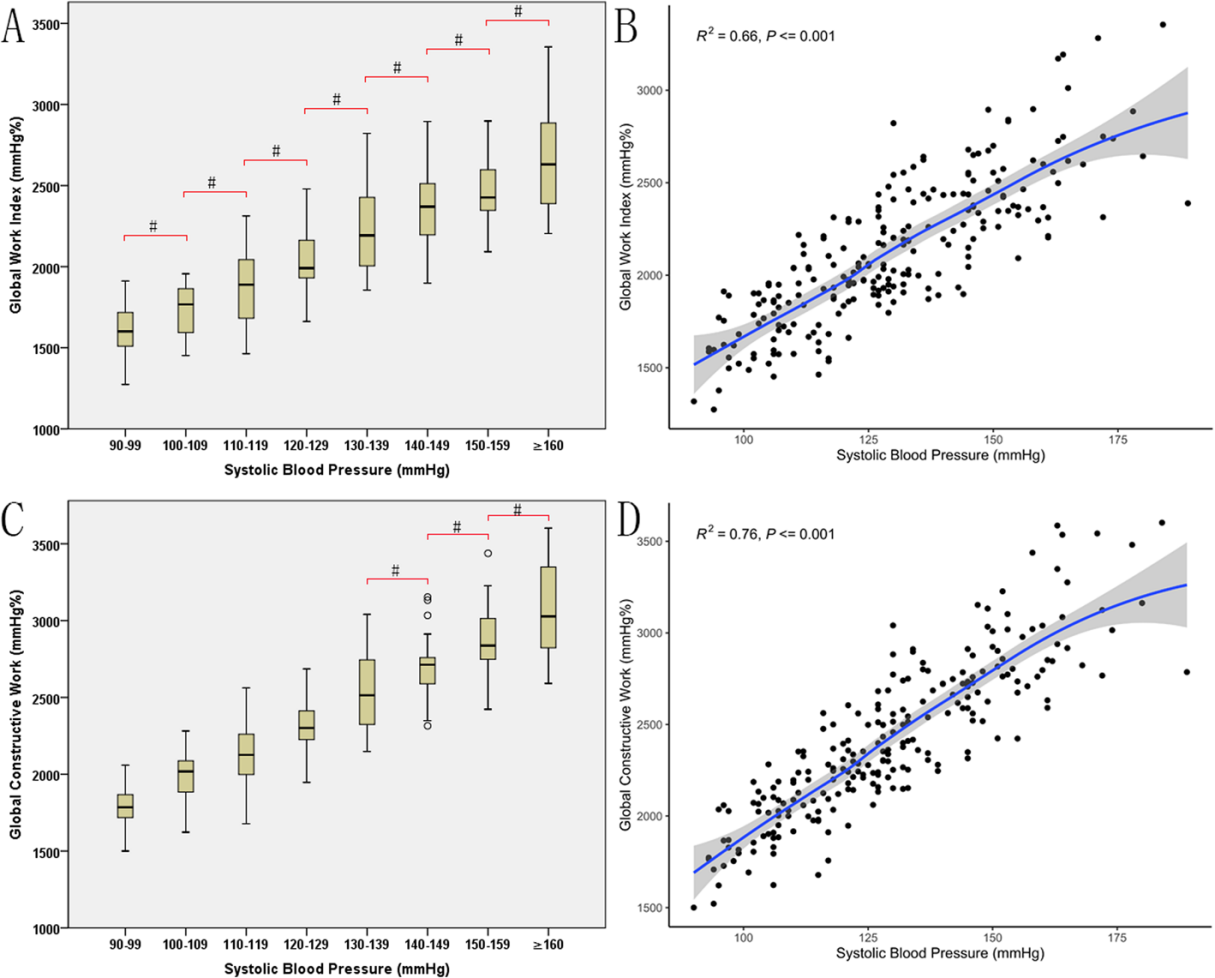
Distribution of GWI and GCW values (**A**, **C**) and association of GWI and GCW with SBP (**B**, **D**). The curves were obtained from locally weighted regression. GWI, global work index; GCW, global constructive work; SBP, systolic blood pressure. ^#^*P* > 0.002, no significant difference

EF, GLS and MW indices grouped by SBP are presented in Table 2. No significant differences were detected for EF and GLS among SBP-groups. When SBP ranged from 90 to 189 mmHg, GWI and GCW tended to increase linearly with the afterload. Significant differences were noted among the SBP-groups for GWI and GCW. For GWI, the majority of the statistical comparisons of the differences at each SBP-group were significant with the exception of the comparisons for every two adjacent groups. For GCW, the majority of the differences between groups were statistically significant with the exception of the comparisons between the SBP 130–139 mmHg and 140–149 mmHg subgroups, the SBP 140–149 mmHg and 150–159 mmHg subgroups and the SBP 150–159 mmHg and SBP ≥ 160 mmHg subgroups (Table 2, Fig. 2).

**Table 2:** EF, GLS and MW indices grouped by SBP

parameters	SBP-groups(mmHg)							
	90–99	100–109	110–119	120–129	130–139	140–149	150–159	≥ 160
EF	63 ± 4	64 ± 4	64 ± 5	65 ± 4	65 ± 5	65 ± 5	64 ± 6	63 ± 5
GLS	-20 ± 2	-20 ± 1	-20 ± 2	-20 ± 1	-21 ± 2	-20 ± 1	-20 ± 2	-19 ± 2
MW indices								
GWI (mmHg%)	1599 ± 182	1743 ± 150	1883 ± 228	2049 ± 182	2223 ± 260	2364 ± 233	2481 ± 211	2690 ± 342
95%CI	1242–1956	1449–2038	1436–2329	1691–2406	1713–2733	1906–2821	2067–2895	2021–3360
GWC (mmHg%)	1792 ± 163	1985 ± 153	2143 ± 200	2329 ± 164	2523 ± 247	2706 ± 191	2868 ± 241	3080 ± 321
95%CI	1471–2112	1684–2285	1752–2535	2007–2650	2039–3007	2332–3080	2395–3341	2451–3708
GWW (mmHg%)	42 ± 14	53 ± 23	54 ± 26	63 ± 20	76 ± 33	85 ± 30	88 ± 31	125 ± 43
95%CI	14–71	8–98	3–105	25–102	11–141	26–143	27–148	40–209
GWE (%)	97(96–98)	97(96–98)	97(96–98)	97(96–97)	97(96–97)	96(95–97)	96(95–97)	95(94–96)
95%CI	94–99	94–99	94–99	95–99	93–99	93–99	94–98	92–98

Data are expressed as mean ± SD or as median (interquartile range)

*EF* ejection fraction; *GLS* global longitudinal strain; *MW* myocardial work; *SBP* systolic blood pressure; *CI* confidence interval; *GWI* global work index; *GCW* global constructive work; *GWW* global wasted work; *GWE* global work efficiency

**Fig. 3:**
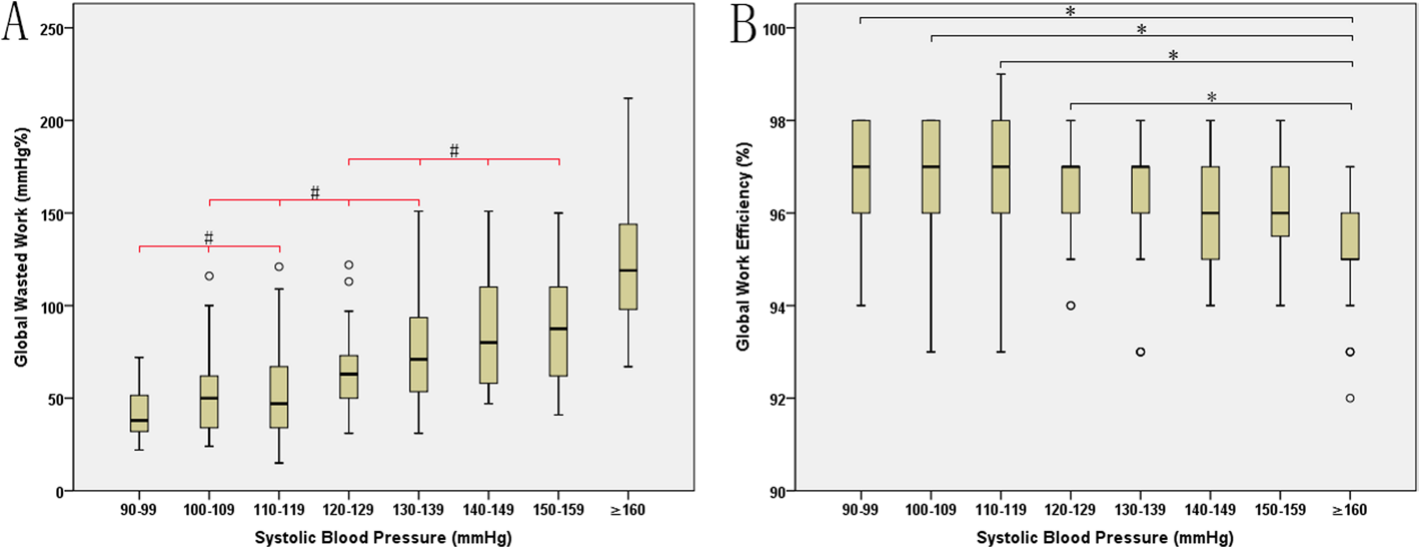
Distribution of GWW (**A**) and GWE (**B**) values with SBP. GWW, global wasted work; GWE, global work efficiency; SBP, systolic blood pressure. ^#^*P* > 0.002, no significant difference. *P < 0.002, significant difference

Overall, GWW tended to rise with the increase of SBP, but not all of the differences in GWW were significant at each SBP group. The differences among the SBP 90–99 mmHg, 100–109 mmHg and 110–119 mmHg subgroups were not statistically significant. Non-significant differences were also noted among the SBP 100–109 mmHg, 110–119 mmHg, 120–129 mmHg and 130–139 mmHg subgroups. Similarly, non-significant differences were noted among the SBP 120–129 mmHg, 130–139 mmHg, 140–149 mmHg and 150–159 mmHg subgroups. The GWE values remained stable among all SBP groups, with the exception of a slight drop noted when SBP exceeded 160 mmHg (Table 2, Fig. 3).

Intra-observer and inter-observer analyses demonstrated optimal repeatability and reproducibility with regard to the MW parameters (Table 3).

**Table 3:** repeatability and reproducibility of MW parameters

variables	Value_1_	Value_2_	Bias_2-1_	*P-*value
Intra-observer				
GWI (mmHg%)	2090 ± 454	2078 ± 457	-13 ± 84	0.350
GCW (mmHg%)	2404 ± 503	2395 ± 507	-9 ± 102	0.583
GWW (mmHg%)	76 ± 34	79 ± 37	3 ± 24	0.492
GWE (%)	96 (95–97)	96 (95–97)	0 (-1–1)	0.826
Inter-observer				
GWI (mmHg%)	2090 ± 454	2071 ± 440	-19 ± 90	0.179
GCW (mmHg%)	2404 ± 503	2375 ± 493	-29 ± 97	0.066
GWW (mmHg%)	76 ± 34	77 ± 38	1 ± 27	0.733
GWE (%)	96 (95–97)	96 (95–97)	0 (-1–1)	0.472

Data are expressed as mean ± SD or as median (interquartile range)

*MW* myocardial work; *GWI* global work index; *GCW* global constructive work; *GWW* global wasted work; *GWE* global work efficiency

## Discussion

MW is derived from pressure–volume or pressure-length loops and it has been investigated for more than 40 years [14–17]. MW assessment was initially measured invasively during cardiac catheterization, which limited its widespread use in clinical practice. Russell et al. introduced recently a method of PSL (in mmHg%) for calculating MW non-invasively, which involved the combination of STE with LVP as estimated from brachial artery cuff pressure [1]. The accuracy of the novel method has been validated by subsequent studies [2–4]. A number of clinical researches have been performed within the last two years, since this technique was commercially available. To date, MW has been investigated with regard to cardiac resynchronization therapy (CRT) [18–23], in the diagnosis of different categories of coronary heart disease [6, 24–28] and in the evaluation of cardiomyopathy [29–33]. In addition, MW can also be used to predict and evaluate therapeutic effects [34–38].

However, due to the lack of accurate reference values used for the MW indices, this method is limited to clinical research but cannot be used for routine clinical examination. Previous studies have obtained reference values of MW grouped by gender or age [7–9]. However, correlations between MW and demographic variables have been investigated, showing the absence of a strong dependence of MW indices on age or gender [7, 8]. Multivariable analysis revealed significant correlations only with SBP for MW parameters [7]. Morbach et al. observed an upward shift of GCW and GWW with advancing age [9]. This finding can be explained when considering the increase of SBP with increased age, even if the SBP remains in the normal range. The present study is the first to measure MW reference values using SBP as a grouping standard. According to our results, GWI and GCW varied greatly according to the different afterload conditions. Therefore, it is unreasonable to assess whether the MW of a given patient is in the normal range in the absence of SBP. For one thing, some patients with myocardial dysfunction will be incorrectly mistaken as normal subjects if the examination results are interpreted using the normal reference ranges previously provided by the EACVI NORRE study [7] for all the patients without taking SBP into account. For example, the normal lowest value of GWI in men was set as 1,270 mmHg%. However, according to our results, this standard was set too low for patients with SBP higher than 100 mmHg, which can lead to misinterpretation of the examinations and result in false negative conclusions. A similar finding was noted for GCW. For another, certain studies have used PSLs to predict myocardial dysfunction and obtained the optimal cut-off values but not considered the effect of SBP on MW [6, 26]. For example, the optimal cutoff for GWI was established as 1,810 mmHg% to predict significant CAD [26]. However, according to our results, it can be normal if GWI is less than 1,810 mmHg% as long as the SBP is low. Clearly, a low GWI due to low SBP can be mistaken for cardiac abnormality resulting in a false positive result. This reason may account for the low diagnostic specificity in that study. Therefore, the critical effect of afterload on MW cannot be ignored during clinical research or diagnosis and is required to make a reasonable judgment on the myocardial function.

Normal reference values have been reported for GWI and GCW and these indices correlated positively with SBP [7–9, 39]. However, no studies have been conducted with regard to the reference values of MW when SBP is above normal. The changes in these indices following increased afterload have not been fully investigated. It is well known that a large percentage of heart disease patients present with hypertension. Therefore, the afterload-dependent reference values are required in this subset of patients for further interpretation. According to our results (SBP of 140–189 mmHg), LVEF and GLS values were preserved in hypertensive subjects without myocardial remodeling and GWI and GCW exhibited a linear association with SBP (Fig. 2). However, this finding may not apply to population with much higher blood pressure. The results demonstrated that the LV myocardium may function at higher energy levels against the increased afterload to preserve LV contractility during the compensatory phase. It is interesting to note that the results from the exercise stress echocardiography confirmed our findings. Healthy subjects demonstrated increased GWI with elevated SBP during exercise, whereas in patients with inducible ischemia, GWI did not increase and MWI was decreased in the affected segments [25, 40, 41]. True myocardial contractility was more likely to be detected under high afterload. The present study was the first to provide work reference for heart disease patients with hypertension. The potential ability to detect myocardial dysfunction under different loading conditions can be employed and assessed in future studies.

The present study demonstrated that GWE values remained constant across all SBP-groups since almost a proportional relationship was noted in both GCW and GWW. Since GWE was not affected by afterload [7, 40, 41] and had a stable reference value in all healthy subjects, it may be more suitable than other parameters as a diagnostic index of myocardial impairment. In some studies, it has been proved that GWE is the best predictor of LV myocardial contractile performance in all MW parameters [27, 28]. However, additional work is required to assess its efficacy in other types of heart disease.

This study has some limitations. Firstly, this was a single-center study including a limited sample size, which may not be sufficient to provide particularly accurate reference values for MW parameters. However, the present study highlighted the importance of afterload in evaluating MW by examining the association between these two parameters and emphasizing on the influence of afterload. This finding cannot be ignored in the clinical research or diagnosis and is required in order to make a reasonable judgment on the myocardial function. Secondly, the present study did not include subjects with SBP > 190 mmHg. Therefore, we could not provide the MW reference and establish the variation trend of MW with SBP in that population. Thirdly, it should be noted that the use of PSL did not provide a direct measure of MW, but rather an index of this parameter due to pressure rather than wall stress being assessed in the method.

## Conclusions

The amount of MW but not the work efficiency varied greatly according to the different afterload. This finding cannot be ignored during clinical research or diagnosis and is required to make a reasonable judgment on the myocardial function.

## Supplementary Information

**Additional file 1.** Data.

## Data Availability

All data generated or analysed during this study are included in this published article (and its supplementary information files).

## Abbreviations

PSL: Pressure-strain loop
MW: Myocardial work
SBP: Systolic blood pressure
GWI: Global work index
GCW: Global constructive work
GWW: Global wasted work
GWE: Global work efficiency
PET: Positron emission tomography
LS: Longitudinal strain
MWI: Myocardial work index
LVP: Left ventricular pressure
LV: Left ventricular
CTA: Coronary tomography angiography
2D: 2-Dimensional
TTE: Transthoracic echocardiography
STE: Speckle tracking echocardiography
EF: Ejection fraction
CW: Constructive work
WW: Wasted work
WE: Work efficiency
SD: Standard deviation
CI: Confidence interval
ANOVA: Analysis of variance
CRT: Cardiac resynchronization therapy
